# Ferritin Nanoparticle Delivery of the E2 Protein of Classical Swine Fever Virus Completely Protects Pigs from Lethal Challenge

**DOI:** 10.3390/vaccines12060629

**Published:** 2024-06-05

**Authors:** Dailang Zhong, Zhanhao Lu, Yu Xia, Hongxia Wu, Xinyu Zhang, Mingzhi Li, Xin Song, Yanjin Wang, Assad Moon, Hua-Ji Qiu, Yongfeng Li, Yuan Sun

**Affiliations:** 1 State Key Laboratory for Animal Disease Control and Prevention, Harbin Veterinary Research Institute, Chinese Academy of Agricultural Sciences, Harbin 150069, China; hsrzlxcrb2021zdl@163.com (D.Z.); luzhanhao106@163.com (Z.L.); xy10841228@163.com (Y.X.); whx450650@163.com (H.W.); zxy2442@163.com (X.Z.); lmz824925@163.com (M.L.); 82101231361@caas.cn (X.S.); wangyanjin1996@126.com (Y.W.); 2021y90100029@caas.cn (A.M.); qiuhuaji@caas.cn (H.-J.Q.); 2 School of Animal Science and Technology, Henan Institute of Science and Technology, Xinxiang 453003, China; 3 School of Life Science and Engineering, Foshan University, Foshan 528225, China

**Keywords:** classical swine fever virus, E2 protein, ferritin, self-assembled nanoparticles, cell-mediated immune responses, neutralizing antibodies

## Abstract

Classical swine fever (CSF), caused by the classical swine fever virus (CSFV), results in significant economic losses to the swine industry in many countries. Vaccination represents the primary strategy to control CSF and the CSFV E2 protein is known as the major protective antigen. However, the E2 protein expressed or presented by different systems elicits distinct immune responses. In this study, we established a stable CHO cell line to express the E2 protein and delivered it using self-assembled ferritin nanoparticles (NPs). Subsequently, we compared the adaptive immune responses induced by the E2-ferritin NPs and the monomeric E2 protein produced by the CHO cells or a baculovirus expression system. The results revealed that the NP-delivered E2 protein elicited higher titers of neutralizing antibodies than did the monomeric E2 protein in pigs. Importantly, only the NP-delivered E2 protein significantly induced CSFV-specific IFN-γ-secreting cells. Furthermore, all the pigs inoculated with the E2-ferritin NPs were completely protected from a lethal CSFV challenge infection. These findings demonstrate the ability of the E2-ferritin NPs to protect pigs against the lethal CSFV challenge by eliciting robust humoral and cellular immune responses.

## 1. Introduction

Classical swine fever (CSF) is an acute and fatal epidemic disease caused by the classical swine fever virus (CSFV), characterized by fever, anorexia, ataxia, and respiratory problems as its main clinical signs [1,2]. It is listed as one of the notifiable pig diseases by the World Organization for Animal Health (WOAH). CSF outbreaks were first documented in the United States in 1860 [3]. In the 20th century, CSF became endemic in many countries and regions, resulting in significant economic losses to the pig industry due to its high morbidity and mortality [4]. To prevent CSF, various vaccines have been developed and applied, while C-strain, a live lapinized attenuated vaccine developed in China, is the most widely used [5]. Alternative vaccines consist of genetically engineered formulations based on the E2 protein of CSFV. Following the implementation of vaccine immunization and strict culling strategies, many regions and countries have successfully eradicated CSF. However, CSF remains endemic in Asia, South and Central Americas, and the Caribbean [6]. After a 26-year absence in Japan, CSF reemerged, and CSFV was detected in both domestic pigs and wild boar, highlighting the complexity of CSFV epidemiology today [7]. Currently, the emergence of the moderately virulent genotype 2 CSFV strains has substituted the previously dominant highly virulent genotype 1 ones [8,9,10,11], thereby posing a greater challenge to the eradication of CSF.

The widespread application of the C-strain vaccination has effectively controlled CSF in China, leading to the absence of large-scale outbreaks in recent years [12]. However, C-strain does not allow differentiating infected from vaccinated animals (DIVA) by serological methods [13]. To enhance the prevention and control of CSF and better preparedness for its eradication, there is an urgent need for a DIVA vaccine that has a comparable efficacy with C-strain. Since the CSFV E2 protein is a major protective antigen capable of inducing neutralizing antibodies (NAbs) [14], subunit vaccines targeting the E2 protein are compatible with DIVA [14,15,16]. Nonetheless, subunit vaccines require a certain period to induce immune responses and cannot provide protection as early as the C-strain vaccine [17]. Therefore, there is a demand for safe and efficacious vaccines that combine both DIVA characteristics and rapid protection.

Different from the attenuated vaccine with the risk of virulence reverse, subunit vaccines are composed of immunogenic peptides, proteins, or polysaccharides derived from the pathogen [18]. However, subunit vaccines typically exhibit limited immunogenicity and protection [19,20]. Although adjuvants can be used to enhance immune responses, subunit vaccines without a suitable delivery platform often require an extended period to induce protective immunity. Consequently, the efficacy of subunit vaccines heavily relies on the utilization of a suitable vaccine delivery platform [21]. Nanoparticles (NPs) efficiently present antigens through various strategies, including chemical modification or gene fusion [22]. In the context of non-fusion expression, NPs are frequently developed as a universal vaccine platform [23]. For example, different antigens can be bound onto the surface of NPs using the SpyCatcher/SpyTag technology derived from *Streptococcus pyogenes* by forming an isopeptide bond [24]. In this context, ferritin from *Helicobacter pylori* was selected to present the antigen [25]. The SpyCatcher/SpyTag coupling technology was utilized to bind ferritin and the E2 protein instead of fusing them directly. This strategy was selected to achieve elevated levels of protein expression [26,27].

Since CHO cells are extensively utilized for glycoprotein antigen production, ferritin NPs have shown remarkable potential in augmenting antigen immunogenicity. In this study, we endeavored to establish a stable CHO suspension cell line for E2 protein expression and then constructed E2-ferritin NPs via the SpyCatcher/SpyTag technology. Finally, we evaluated its immunogenicity and protective efficacy in pigs.

## 2. Materials and Methods

### 2.1. Cells and Virus

The Expi-CHO-S cell line (A37785, Thermo Fisher Scientific, Waltham, MA, USA) was cultured in CHOGrow CD2 serum-free medium (BesalMedia, Shanghai, China) supplemented with L-glutamine (BesalMedia, Shanghai, China). The human embryonic kidney-derived HEK293T cells (CL-0005, Procell, Wuhan, China) and the porcine kidney-derived PK-15 cells (CL-0187, Procell, Wuhan, China) were grown in Dulbecco’s modified Eagle medium (DMEM) (Gibco, Carlsbad, CA, USA) supplemented with 10% fetal bovine serum (FBS) (Gibco, Carlsbad, CA, USA). The highly virulent CSFV Shimen strain (CSFV-SM) (GenBank no. AF092448.2) used as the challenge virus was propagated in PK-15 cells.

### 2.2. Design and Construction of Plasmids

**Figure 1:**
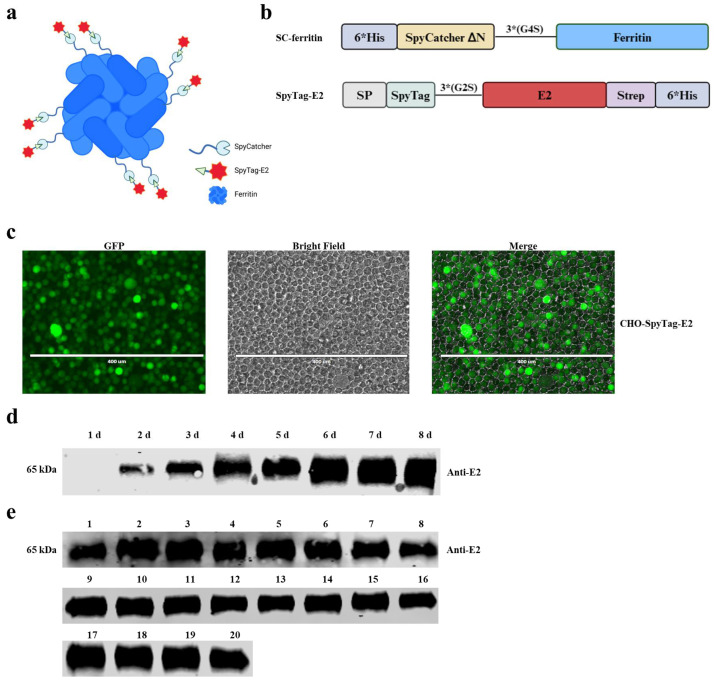
Molecular design of the E2-ferritin nanoparticles (NPs) and generation of the CHO-SpyTag-E2 cell line. (**a**) The schematic diagram depicting the E2-ferritin NPs. Each ferritin subunit is linked to a copy of the E2 protein, all E2 proteins being positioned on the surface of the NPs. (**b**) The schematic diagram illustrating the vaccine design. 6 × His-tagged SpyTag-E2 and SpyCatcher-ferritin, produced by the Expi-CHO-S cells and *E. coli* BL21 (DE3), respectively. (**c**) The generation of the CHO-SpyTag-E2 cell line. The ZsGreen gene was integrated into the CHO cells by the recombinant lentivirus, resulting in the expression of the green fluorescent protein (GFP). (**d**) The SpyTag-E2 protein accumulated in the supernatants of the CHO-SpyTag-E2 cells. The anti-E2 monoclonal antibody was used to verify the accumulation process of SpyTag-E2 from day 0 to day 8 by Western blotting. (**e**) The stable expression of the SpyTag-E2 protein in the CHO-SpyTag-E2 cells after 20 consecutive passages (numbers 1–20 represented the tested samples for each generation). The anti-E2 monoclonal antibody was used to validate the consistent expression of SpyTag-E2 in each passage of the CHO-SpyTag-E2 cells by Western blotting.

The plasmid pCold-I was used to express the SpyCatcher-ferritin NPs (SC-ferritin NPs) in *Escherichia coli* (*E. coli*) BL21 (DE3). The encoding sequence of SC-ferritin, comprised of ΔN-SpyCatcher (GenBank: MH425515) and ferritin (UniProt: Q9ZLI1), was codon-optimized for *E. coli*, and synthesized by BHI. Subsequently, it was cloned into pCold-I, incorporating an N-terminal 6 × His tag. The ΔN-SpyCatcher sequence was fused to the N-terminus of ferritin, facilitating the presentation of the ΔN-SpyCatcher protein on the surface of the NPs (Figure 1a,b). The resulting SC-ferritin-encoding sequence was cloned into the pCold-I vector by homologous recombination.

The modified pCold-I expression plasmid containing the ΔN-SpyCatcher-ferritin fusion sequence was introduced into the *E. coli* BL21 (DE3) competent cells. Subsequently, a single positive colony was transferred into 10 mL of LB medium supplemented with 50 μg/mL ampicillin. The culture was incubated overnight at 37 °C with shaking at 220 rpm.

The transmembrane region of the CSFV E2 protein (GenBank: ANI21172.1) was deleted. Codon-optimized E2 for the Chinese hamster was synthesized by BGI. It was then cloned into the pLVX-IRES-ZsGreen plasmid with an N-terminal Kozak consensus sequence, a GT6 signal peptide for protein secretion, a 13-amino-acid SpyTag for conjugation to SpyCatcher, and a C-terminal 6 × His tag. The resulting plasmid was named pLVX-SpyTag-E2. It was then extracted from *E. coli* DH5α using the EndoFree Maxi plasmid kit (Tiangen, Beijing, China) following the manufacturer’s protocols.

### 2.3. Expression and Purification of the SC-Ferritin NPs

The activated *E. coli* BL21 (DE3) transformed with pCold-Fe were inoculated into 1 L of LB medium supplemented with 50 μg/mL ampicillin and cultured at 37 °C with shaking at 150 rpm. Upon reaching an OD_600nm_ range of 0.6 to 0.8, protein expression was induced by adding isopropyl thiogalactoside (IPTG) to a final concentration of 1 mM. The induction proceeded at 16 °C for 16 to 20 h with agitation at 110 rpm. Following the induction, the bacterial cultures were harvested by centrifugation at 8000× *g* and 4 °C for 3 min. The resulting precipitate was resuspended in 50 mM PBS at pH 7.3 and then lysed via sonication. The lysate was further centrifuged at 12,000× *g* and 4 °C for 20 min, and the supernatant was filtered by a 0.45 µm Millex HV filter (Millipore, Billerica, MA, USA). Subsequently, the concentrated supernatant was purified using Ni Sepharose High Performance (Cytiva, Marlborough, MA, USA) in a 12 mL affinity chromatography column (Byotime, Shanghai, China). The target protein was eluted with 50 mM PBS (pH 7.3) containing 300 mM imidazole. The eluted samples were subjected to evaluation by SDS-PAGE. The buffer was exchanged with 50 mM HEPES at pH 7.3 and 300 mM NaCl. The quantification of the SC-ferritin NPs was conducted using a bicinchoninic acid (BCA) kit (Solarbio, Beijing, China) and stored at 4 °C for subsequent studies.

### 2.4. Establishment and Characterization of the CHO-SpyTag-E2 Cell Line

The SpyTag-E2 protein was expressed by the Expi-CHO-S cells. To generate the recombinant lentivirus for establishing the CHO-SpyTag-E2 cell line, a mixture containing 10.5 μg of pLVX-SpyTag-E2, 7 μg of pSPAX2, and 3.5 μg of pMD2.G was transfected into the HEK293T cells using X-tremeGENE HP DNA (Sigma-Aldrich, St. Louis, MO, USA). The lentivirus was harvested and concentrated using the Amicon Ultra-15 Centrifugal Filter Unit (Millipore, Billerica, MA, USA) after 48 h. Subsequently, 30 mL of the Expi-CHO-S cells (a million cells per mL) were infected with the lentivirus, and 30 μL of polybrene (G-CLONE, Beijing, China) was added. The infected cells were centrifuged for 5 min at 500× *g* at room temperature, and the cell culture volume was increased to 60 mL after 3 days. The cells were continuously suspended and cultured at a million cells per mL for 8 days. The supernatant was taken daily for Western blotting to assess the expression kinetics and determine the optimal time for protein collection. The CHO-SpyTag-E2 suspension cell line was continuously passaged for 20 generations. Finally, the cell line was cryopreserved in a solution containing 10% DMSO and 90% complete culture medium in a liquid nitrogen.

### 2.5. Expression and Purification of SpyTag-E2

The CHO-SpyTag-E2 cells continuously expressed protein at a density of a million cells per mL in a 300 mL culture medium. After 5 days of culture, cells were harvested by centrifugation at 8000× *g* and 4 °C for 10 min, and the resulting supernatant was filtered by a 0.45 µm Millex HV filter. The concentrated supernatant was then subject to purification using Ni Sepharose High Performance in a 30 mL affinity chromatography column (Byotime, Shanghai, China). The target protein was eluted with a buffer comprising 50 mM PBS at pH 7.3, containing 300 mM imidazole, and verified by Western blotting. The buffer was exchanged with 50 mM HEPES at pH 7.3 and 300 mM NaCl. The SpyTag-E2 was quantified using a bicinchoninic acid (BCA) kit and stored at 4 °C for the following studies.

### 2.6. Generation and Purification of the E2-Ferritin NPs

The SC-ferritin and SpyTag-E2 at a 1:4 molar ratio were incubated overnight at 4 °C in 50 mM pH 7.3 HEPES and 300 mM NaCl. To isolate the E2-ferritin NPs from excess SpyTag-E2 and SC-ferritin NPs, size exclusion chromatography (SEC) was conducted using a pre-equilibrated Superose 6 Increase 10/300 GL gel filtration column (Cytiva, Marlborough, MA, USA) with 50 mM pH 7.3 HEPES and 300 mM NaCl. The protein solutions from all peaks were collected for Western blotting analysis. The E2-ferritin NPs were visualized using negative staining transmission electron microscopy (TEM), concentrated, and stored at 4 °C for subsequent immunization.

Endotoxins in the E2-ferritin NPs were eliminated using the ToxinEraser endotoxin removal kit (Genscript, Nanjing, China).

### 2.7. Measurement of Particle Size

The size distribution of the SC-ferritin and E2-ferritin NPs was measured by dynamic light scattering (DLS, Zetasizer Nano ZS-90, Malvern Panalytical, Malvern, UK).

### 2.8. Immunization/Challenge Experiment in Pigs

Thirteen 4-week-old pigs were randomly allocated into four groups: group A (*n* = 4), group B (*n* = 4), group C (*n* = 3), and group D (*n* = 2). Groups A and B received intramuscular injections of 50 µg of the E2-ferritin NPs or SpyTag-E2, respectively. Group C was intramuscularly injected with a commercial subunit vaccine of CSF (produced and purified from the baculovirus expression system, each containing 50 µg of the CSFV E2 protein, CV-E2), while group D was intramuscularly injected with PBS as the control group. A second immunization was administered at 14 dpi. Each pig was challenged with 10^5^ TCID_50_ of CSFV-SM at 28 dpi. Blood samples were collected, and sera were separated at 3, 7, 10, and 14 days post-challenge (dpc). The daily measurements of rectal temperatures and clinical scores were recorded, with clinical scoring based on five clinical parameters including physical tension, walking, appetite, defecation, and eyes/conjunctiva (normal = 0; mild = 1; obvious = 2; severe = 3) [22]. When animals exhibited one of the following symptoms: complete loss of appetite, severe convulsions, imminent death marked by a body temperature drop below 37.5 °C, inability to stand, or significant weight loss, we considered it to have reached the humane endpoint, and euthanasia was performed. At 14 dpc, all the pigs were euthanized, and then the inguinal lymph nodes, mandibular lymph nodes, kidneys, tonsils, and spleens were collected.

### 2.9. Enzyme-Linked Immunosorbent Assay (ELISA)

The sera were tested for the presence of CSFV-specific antibodies using a blocking ELISA kit following the manufacturer’s instructions (CSFV antibody test kit, IDEXX, Westbrook, ME, USA). The IFN-γ, IL-2, and IL-4 concentrations in the serum samples at the indicated time points following immunization were measured using a double-antibody sandwich-ELISA kit (MLBio, Shanghai, China).

### 2.10. Serum/Virus Neutralization Test

The serum samples were subjected to heat inactivation at 56 °C for 30 min. Subsequently, the sera were serially diluted in a two-fold manner, reaching a final dilution of 1:12,800. Then, 100 µL of CSFV-SM (200 TCID_50_) was mixed with 100 µL of the sera at the specified dilution creating a 1:1 ratio mixture of the sera and CSFV-SM. The mixture was incubated for 1 h at 37 °C. Next, the virus/serum mixture was added to PK-15 cells in 96-well plates. After 1 h of adsorption, the mixed solution was discarded, and a DMEM cell culture medium containing 2% FBS was added. The cells were then cultured at 37 °C with 5% CO_2_ for 2 days. The presence of the CSFV-infected cells in each well was determined using an immunofluorescence assay (IFA) as described previously [28]. The titers of NAbs were calculated and expressed as the logarithm of the highest dilution at which infection of 50% PK-15 cells in cultured wells were inhibited from infection.

### 2.11. IFN-γ Enzyme-Linked Immunospot Assay

Peripheral blood mononuclear cells (PBMCs) from peripheral blood treated with heparin (Solarbio, Beijing, China) were collected at 21 and 28 dpi and adjusted to a concentration of 2 million cells/mL by using RPMI-1640 medium containing 10% FBS. Subsequently, 200 μL of RPMI-1640 medium containing 10% FBS was added to each well of pre-coated ELISpot plate (3130-4APW-2, mAb of pIFNγ-I) and incubated for 1 h at room temperature. After discarding the culture medium, 50 μL of the prepared PBMC suspension was added to each well, followed by the addition of 50 μL of the CSFV E2 protein solution (100 μg/mL) to achieve a final concentration of 1 million cells and 5 μg of E2 protein per well. The negative control group was treated with 50 μL of serum-free RPMI-1640 medium, while the positive control group received 2.5 µg/mL PHA. The plate was wrapped in aluminum foil, incubated at 37 °C with 5% CO_2_ for 48 h, and washed with PBS. Then, 100 µL of 0.5 µg/mL biotinylated detection antibody (P2C11 biotin) was added to each well and incubated at room temperature for 2 h, followed by another wash with PBS. Subsequently, 100 μL of streptavidin HPR (diluted at 1:1000) was added to each well, incubated at room temperature for 1 h, and washed with PBS. To visualize the spots, 100 μL of TMB substrate was added to each well. The plate was rinsed with deionized water until the spots no longer diffused. The plate was then dried, and the spots were counted by ISpot Spectrum ELR088IFL. The CSFV-specific IFN-γ-secreting cells in the PBMCs were quantified as the number of spots per million cells.

### 2.12. Real-Time RT-PCR (RT-qPCR)

The total RNAs from anticoagulant samples, and oral, nasal, and rectal swab samples were extracted using the kit (Tiangen, Beijing, China). Subsequently, the cDNA was synthesized in a 20 μL volume using avian myeloblastosis virus reverse transcriptase XL (Tiangen, Beijing, China). The copies of the CSFV genome were quantified using RT-qPCR as described previously [29].

### 2.13. Statistic Analysis

The data were analyzed in GraphPad Prism 8.0.2. Statistical significance was determined at ns, not significant (*p* > 0.05); *, *p* < 0.05; **, *p* < 0.01; ***, *p* < 0.001; ****, *p* < 0.0001.

## 3. Results

### 3.1. Characteristics of the CHO-SpyTag-E2 Cell Line

At 48 h post-transduction of the Expi-CHO-S cells with the recombinant lentivirus, green fluorescence was observed in the suspension cells under a fluorescence microscope, indicating the successful lentivirus transduction of the Expi-CHO-S cells and the integration of the corresponding gene into the cell genome (Figure 1c). Western blotting confirmed the successful expression of the SpyTag-E2 protein in the CHO-SpyTag-E2 suspension cell line, signifying the activation of the SpyTag-E2 secretion expression sequence. The results of 8 consecutive days of expression sample detection showed a continuous increase in protein expression (Figure 1d). However, during the cell culture process, a decrease in cell viability was observed starting on the 6th day of expansion culture, reaching 0% by the 8th day. Therefore, a large quantity of protein could be harvested on the 5th day, and cells could be passaged to maintain protein expression. Considering the sustainable application of the cell line, we chose to collect samples on the 5th day after expansion culture.

After continuous passaging for 20 generations, the secretion and expression of the SpyTag-E2 protein in the supernatant of each generation were assessed by Western blotting. These results confirm that the CHO-SpyTag-E2 suspension cell line can consistently and stably express the SpyTag-E2 protein (Figure 1e).

### 3.2. Structural Characteristics of the E2-Ferritin NPs

**Figure 2:**
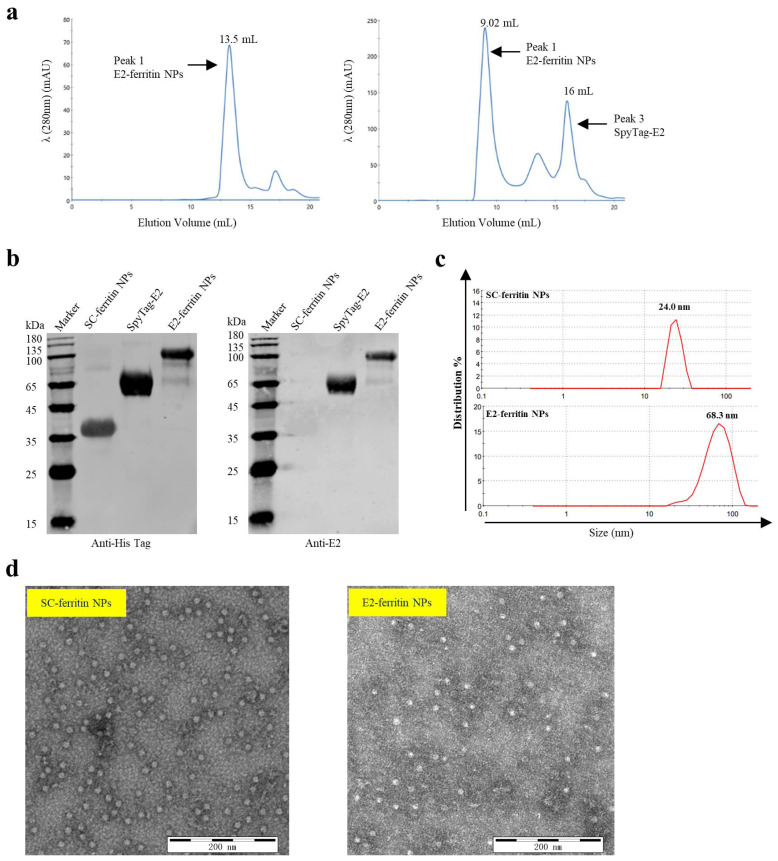
Structural characteristics of the E2-ferritin NPs. (**a**) The size-exclusion chromatography of the SC-ferritin and E2-ferritin NPs. The ultraviolet absorptions at 280 were recorded, revealing a leftward shift in the elution peak of the E2-ferritin NPs, indicative of an increase in particle size and confirming the loading of the E2 protein onto the surface of the ferritin NPs. (**b**) The Western blotting analysis of the SC-ferritin NPs, SpyTag-E2, and E2-ferritin NPs. Both anti-His and anti-E2 antibodies were used to identify the expression and purity of each protein. (**c**) The dynamic light scattering size measurements of SC-ferritin and E2-ferritin NPs. (**d**) The transmission electron microscopy of the self-assembled SC-ferritin and E2-ferritin NPs.

The SpyTag-E2 protein and SC-ferritin NPs were purified individually. Subsequently, we further purified the E2-ferritin NPs using size exclusion chromatography (SEC). The results revealed a significant increase in the size of the E2-ferritin NPs compared with ferritin alone, as evidenced by a substantial forward shift in the SEC elution peak from 13.50 to 9.02 mL (Figure 2a), indicating the successful loading of the E2 protein onto the surface of ferritin. Furthermore, the conjugation of these two proteins was verified by Western blotting. The molecular weights of approximately 35.5 kDa for SC-ferritin NPs, 65 kDa for SpyTag-E2, and 100 kDa for E2-ferritin NPs were observed. The anti-6xHis Tag antibody recognized all three proteins, while the anti-E2 monoclonal antibody reacted specifically with SpyTag-E2 and E2-ferritin NPs, confirming the successful binding of SpyTag-E2 and SC-ferritin NPs (Figure 2b). Dynamic light scattering analysis reveals that the diameter of SC-ferritin NPs measures approximately 24.0 nm, whereas that of E2-ferritin NPs is around 68.3 nm (Figure 2c). This observation emphasizes the conjugation of SpyTag-E2 on the surface of ferritin NPs, thereby contributing to the observed increase in diameter. The structural characterization of the E2-conjugated NPs was performed using negative staining TEM, revealing the successful assembly of the E2-ferritin NPs with abundant protein surrounding the NPs (Figure 2d). Overall, these findings confirm the formation of the E2-conjugated NPs.

### 3.3. The E2-Ferritin NPs Induced High Titers of CSFV-Specific NAbs in Pigs

**Figure 3:**
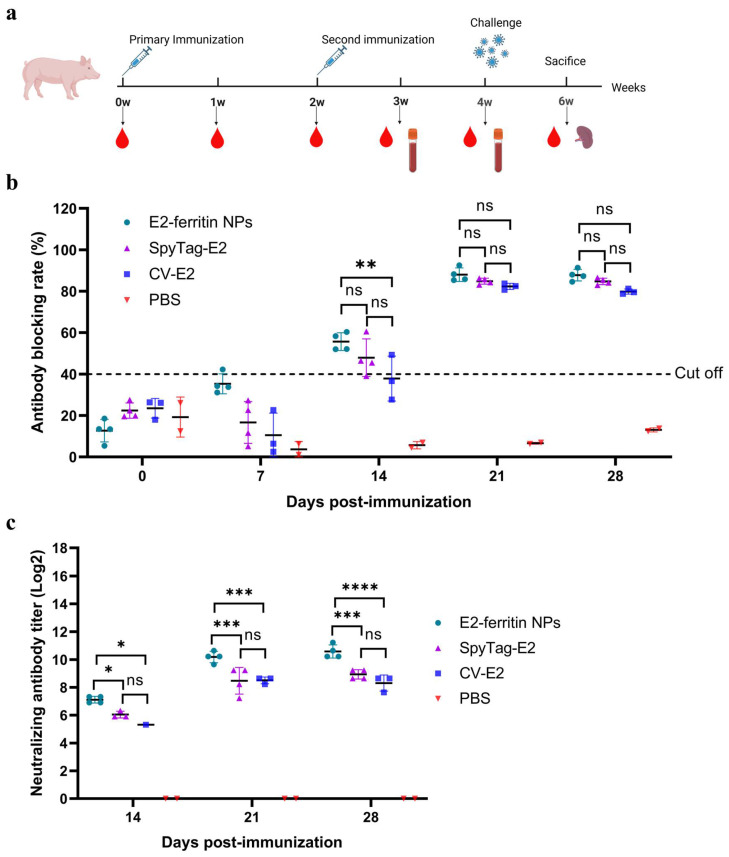
The E2-ferritin NPs induced high-titer CSFV-specific NAbs in the pigs. (**a**) The schematic of pig vaccination. The pigs in each group were vaccinated with different vaccines, and serum samples were collected once a week. At 28 dpi, all the pigs were challenged with 10^5^ TCID_50_ of CSFV-SM and euthanized at 14 dpc. (**b**) The detection of anti-E2 antibodies. The anti-E2 antibodies in the sera of each group at the indicated dpi were checked by an antibody-blocking ELISA. (**c**) NAbs in each group at different dpi. The titers of NAbs were determined as the highest serum dilution that inhibits virus infection and expressed as log2 NAb titers. The data were analyzed using the two-way ANOVA, bars represent the means ± SD; ns, not significant (*p* > 0.05); *, *p* < 0.05; **, *p* < 0.01; ***, *p* < 0.001; ****, *p* < 0.0001.

To assess the efficacy of E2-ferritin NPs in inducing protective immune responses, the pigs were immunized with either 50 μg of SpyTag-E2 or E2-ferritin NPs containing 50 μg of SpyTag-E2. The remaining two groups were immunized with CV-E2 (50 μg E2 protein per dose) or 2 mL of PBS (Figure 3a).

All the serum samples collected from the pigs were tested for the anti-E2 antibodies using a blocking ELISA kit. CSFV-specific antibodies were not detected in the serum samples collected before immunization, indicating that all the pigs were initially CSFV-negative individuals. At 7 dpi, one pig in the E2-ferritin NPs group tested positive for CSFV-specific antibodies. At 14 dpi, the positivity rate of the CSFV-specific antibodies reached 100% in the E2-ferritin NPs group, compared to 75% in the SpyTag-E2 group and 50% in the CV-E2 group (Figure 3b). Notably, at 14, 21, and 28 dpi, the NAb titers of the E2-ferritin NPs group were significantly higher than those of both the SpyTag-E2 group and the CV-E2 group (Figure 3c).

### 3.4. The E2-Ferritin NPs Elicited Robust Cell-Mediated Immune Responses in Pigs

To evaluate the advantages of the E2-ferritin NPs in inducing specific cellular immunity, we quantified the concentrations of IFN-γ, IL-2, and IL-4 in sera collected from each immune group. Additionally, PBMCs were isolated from anticoagulated blood for the detection of IFN-γ-producing cells using ELISpot.

**Figure 4:**
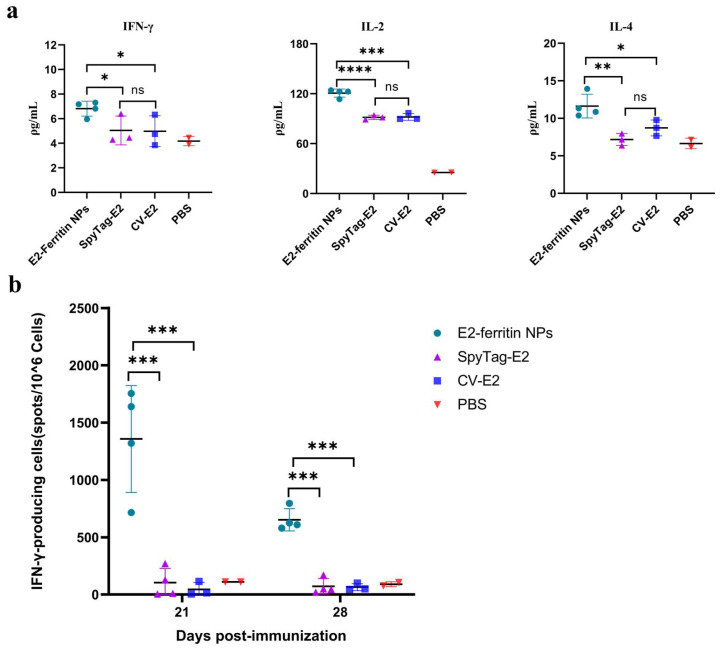
The E2-ferritin NPs elicited robust cell-mediated immune responses in the pigs. (**a**) Cytokines in the sera of the immunized pigs. IL-2, IFN-γ, and IL-4 in the serum samples from each group at 28 dpi were measured using a double-antibody sandwich-ELISA. (**b**) The number of CSFV-specific IFN-γ-secreting cells from different groups. The CSFV-specific IFN-γ-secreting cells in the PBMCs from the immunized pigs at 21 or 28 dpi were quantified as the number of spots per 10^6^ cells using ELISpot. The data were analyzed using the one-way ANOVA, bars represent the means ± SD; ns, not significant (*p* > 0.05); *, *p* < 0.05; **, *p* < 0.01; ***, *p* < 0.001; ****, *p* < 0.0001.

The ELISA results showed that the concentrations of Th1 cytokines IFN-γ and IL-2 of the E2-ferritin NPs group were significantly higher than those of the SpyTag-E2 group (*p* < 0.05 and *p* < 0.0001, respectively) and the CV-E2 group (*p* < 0.05 and *p* < 0.001, respectively) at 28 dpi. However, there was no significant difference between the SpyTag-E2 group and the CV-E2 group. Regarding Th2 cytokines, the IL-4 concentration in the E2-ferritin NPs group was also significantly higher than those in the SpyTag-E2 group and the CV-E2 group (*p* < 0.01 and *p* < 0.05, respectively) (Figure 4a).

PBMCs were isolated from the peripheral blood of the immunized pigs at 21 and 28 dpi in each group. The ELISpot results revealed that the average numbers of spots produced per million cells in the E2-ferritin NPs group after stimulation with the E2 protein were significantly higher than that in the SpyTag-E2 group and the CV-E2 group (*p* < 0.001). These findings indicate the development of CSFV-specific cell-mediated immunity in the pigs (Figure 4b).

### 3.5. The E2-Ferritin NPs Conferred Complete Protection of Pigs against Lethal CSFV Challenge

**Figure 5:**
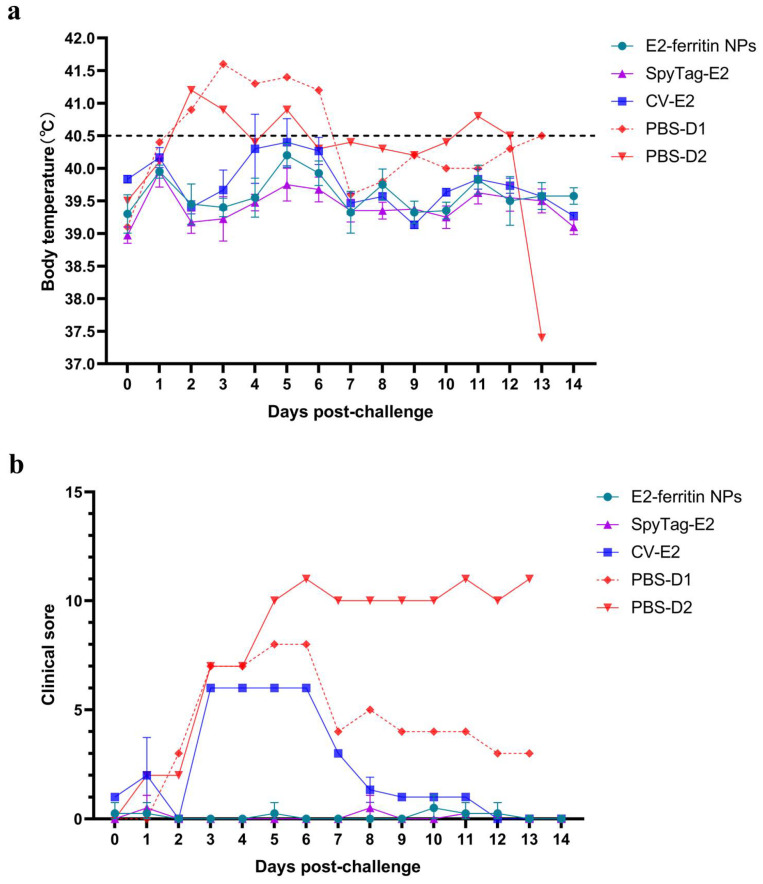
The E2-ferritin NPs conferred complete protection of the pigs from the lethal CSFV challenge. (**a**) The rectal temperatures of the pigs following the challenge with 10^5^ TCID_50_ of CSFV-SM. (**b**) Clinical scores based on the five typical clinical signs of CSF.

Both the E2-ferritin NPs and SpyTag-E2 induced complete protection against CSFV in the pigs in vivo. After the lethal CSFV challenge, the PBS group showed acute fever (40.5–42.1 °C) (Figure 5a). Additionally, typical clinical signs caused by CSFV include mild diarrhea, chills, loss of appetite, and cyanosis. At 13 dpc, the pigs in the PBS group reached the humane endpoint and were euthanized (Pig D2 displayed all five symptoms, whereas pig D1 exhibited complete loss of appetite). However, the CV-E2 group experienced transient fever lasting 1–2 days, accompanied by clinical manifestations persisting for 4 days, followed by a swift resolution (Figure 5b).

**Figure 6:**
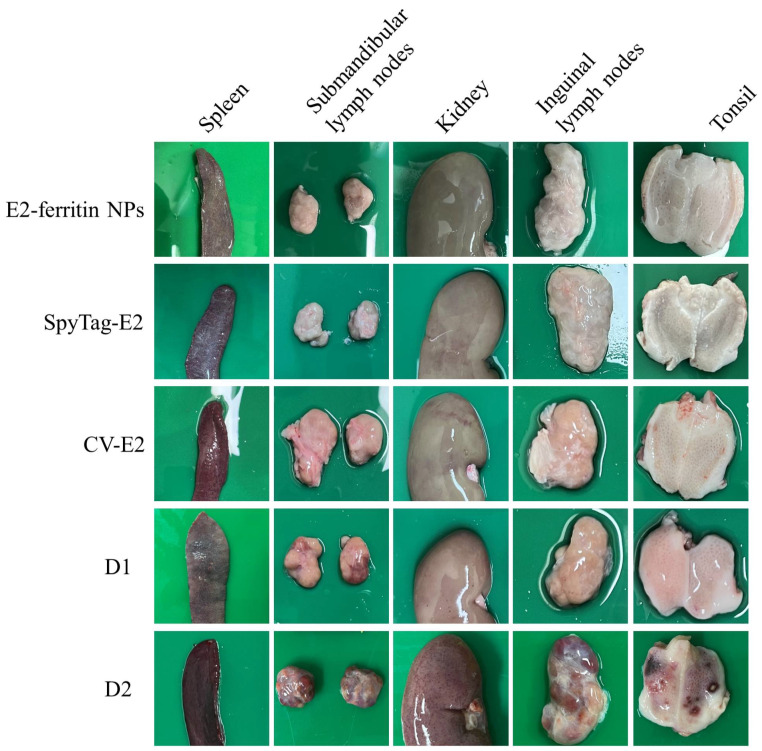
Representative gross pathological manifestations in tissues following CSFV challenge infection. A variety of tissue specimens, including spleens, submandibular lymph nodes, kidneys, inguinal lymph nodes, and tonsils, were collected for gross pathological examination.

**Figure 7:**
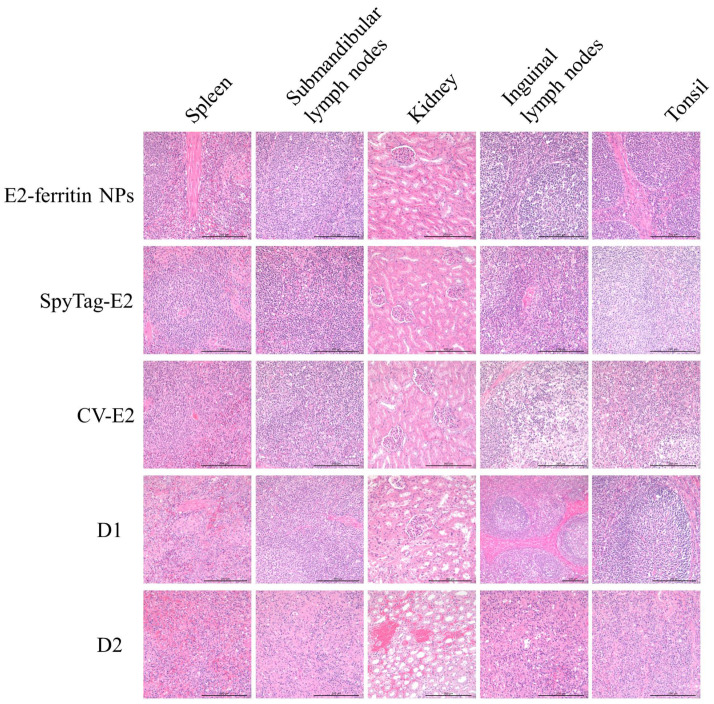
Representative histopathological images of tissue specimens from the spleen, submandibular lymph nodes, kidneys, inguinal lymph nodes, and tonsils using hematoxylin and eosin staining (H&E).

The gross pathological examination also confirmed the protective effect of the E2-ferritin NPs. The examination revealed no significant changes in tissue organs in both the E2-ferritin NPs and the SpyTag-E2 group. The CV-E2 group exhibited slight hemorrhage in the kidneys and tonsils. The PBS group showed severe clinical lesions, including hemorrhagic spots in the kidneys, lymph node enlargement with hemorrhage, and tonsillar bleeding (Figure 6). Also, no histopathological changes were observed both in the E2-ferritin NPs group and the SpyTag-E2 group. The CV-E2 group exhibited minor pathological changes, characterized by a slight reduction in lymphocytes in the lymphoid follicles and paracortical areas of the inguinal lymph nodes and in the tonsils. In contrast, the PBS group displayed severe pathological changes, including blurred intrinsic tissue structures in the inguinal lymph nodes, submandibular lymph nodes, and tonsils; significant atrophy or loss of structure in the white pulp of the spleen; and hemorrhage along with structural damage in the kidneys (Figure 7).

Correspondingly, the pigs immunized with E2-ferritin NPs and SpyTag-E2 did not show viremia. In the PBS group, the viral RNA exhibited a rapid increase, with detectable high copy numbers as early as 3 days post-challenge (dpc). By 7 dpc, viral RNA levels reached 10^4^ copies/mL, culminating in a peak concentration of 10^6^ copies/mL at 10 dpc. In contrast, the CV-E2 immunization group showed lower viremia at 7 dpc and was cleared at 10 dpc (Table 1). We found that none of the individuals in the E2-ferritin NPs and SpyTag-E2 groups exhibited viremia, and the viral RNA was not detected in nasal, oral, and rectal swabs. Conversely, the CSFV gene was detected in all types of swabs of the PBS group. These findings suggest that following the virulent CSFV challenge, the virus was quickly neutralized and cleared in the pigs immunized with E2-ferritin NPs and SpyTag-E2, without any virus excretion (Table 2, Table 3 and Table 4). It is worth noting that the CV-E2 group exhibited peak viremia at 7 dpc, accompanied by minimal virus shedding.

## 4. Discussion

NP-based vaccine platforms have been extensively used in the research and development of various diseases, especially cancer vaccines, encompassing both preventive and therapeutic vaccines [30]. One of the key advantages of NP-based vaccines is their capacity to elicit robust and enduring immune responses [31]. In general, protein antigens are fragile and prone to degradation in the complex humoral microenvironment of the body. This fragility can result in decreased delivery to antigen-presenting cells (APCs) and ineffective immune responses. NP-based delivery platforms offer a solution to this problem [32]. Currently, clinical trials for NP-based vaccines have commenced [33], which indicates the potential application of such vaccines.

The E2 protein of CSFV is the main immunogen that can induce NAbs in pigs [34]. Therefore, it is the target for the development of CSF subunit vaccines [35]. However, E2-based subunit vaccines cannot induce cellular immunity. In the early stages of immunity, C-strain mainly relies on IFN-γ to exert protective effects [36]. This highlights a notable advantage of C-strain compared to subunit vaccines. Therefore, there is an urgent need for a subunit vaccine that can induce cellular immunity to replace C-strain for CSF control. In this study, IFN-γ ELISpot analysis revealed that E2-ferritin NPs can activate a robust T-cell immune response, nearly 500 times stronger than that of conventional subunit vaccines. However, the difference in IFN-γ concentrations in serum was not as substantial as in the ELISpot results. This discrepancy may be due to the activation state of IFN-γ-secreting cells, the half-life of IFN-γ, or the body’s immunoregulatory mechanisms [37].

It has shown that neutralizing antibody titers ≥ 1:32 can effectively counteract CSFV infection [38]. In the present study, we observed that immunization with 50 μg of E2-ferritin NPs proved to be highly effective in eliciting higher titers of NAbs in pigs. At each sampling time point, the NAbs titers in the E2-ferritin NP-immunized group were significantly higher than those in the SpyTag-E2 protein-immunized group. Notably, the NAbs titers of the E2-ferritin NPs group exceeded 1:100 at 14 dpi. Our findings suggest that a single dose of immunization may elicit protective immune responses, but it necessitates further challenge experiments for validation.

In contrast to the subunit vaccine groups, the group immunized with E2-ferritin NPs exhibited high levels of IL-2 and IFN-γ at 28 dpi, indicating that the E2-ferritin NPs can induce cellular immunity. Furthermore, the E2-ferritin NPs induced NAbs at 14 dpi, with an average antibody titer of 1:120. Therefore, the E2-ferritin NPs rapidly induced complete protective immunity against the lethal CSFV challenge, attributed to both humoral and cellular immune responses. Moreover, we observed that nanoparticle-delivered E2 protein from genotype 2 CSFV can induce complete protection against CSFV-SM (CSFV of genotype 1). Additionally, the stimulation of PBMCs from immunized animals with SpyTag-E2 (sequence derived from genotype 2 CSFV) resulted in the activation of a substantial number of CSFV-specific IFN-γ-secreting cells. These results suggested that E2-ferritin NPs may induce complete protection against both genotype 1 and 2 strains of CSFV. However, further evaluation in animal models is necessary to confirm this.

We observed that, under the same immunization regimen, the pigs vaccinated with the CSF subunit vaccine produced by the baculovirus expression system exhibited a transient period of viremia and clinical signs following the lethal challenge. However, these signs quickly subsided. In contrast, such occurrences were not observed in the pigs vaccinated with SpyTag-E2, suggesting that the E2 protein produced by the CHO cell lines may confer superior immunogenicity. This difference may arise from the distinct characteristics of the expressed proteins in insect cells compared to mammalian cells. Insect cells can only modify N-glycosylation into paucimannose or oligomannose structures [39,40]. However, the core immunogenic epitope of the E2 protein domain contains high-mannose and complex/hybrid N-glycosylation modifications [41]. This suggests that the CSFV E2 protein subunit vaccines are better suited for production using the CHO expression system.

NP-based vaccines, as a type of protein-based vaccine, can induce stronger humoral immune responses compared to conventional subunit vaccines. This heightened efficacy is attributed to several key factors. Firstly, NPs are comparable in size to virus particles, facilitating their capture by APCs [42]. Furthermore, NPs possess the unique ability to directly access lymph nodes [43], thereby providing a sustained and potent stimulus to germinal center B cells (GC B cells), leading to the increased frequency of somatic hypermutations and the rapid generation of more specific B cells capable of producing NAbs [44]. Moreover, previous studies have indicated that NPs, upon uptake by dendritic cells, possess a probability of antigen presentation via MHC-I, a phenomenon termed cross-presentation, thereby eliciting cellular immunity [45,46]. In this study, we observed that the E2-ferritin NPs elicited a potent T cell immune response, possibly attributed to the stability of the nanoparticles in vivo. Ferritin, as an iron-binding protein, exhibits remarkable stability under physiological conditions [47]. Consequently, it may sustain the prolonged stimulation of memory cells after the second immunization. However, this stimulation is likely regulated by the body’s immune modulation mechanisms, as indicated by the substantial decline in the number of CSFV-specific IFN-γ-secreting cells in PBMCs between 7 and 14 days after secondary immunization. The precise mechanisms underlying this phenomenon warrant further investigation.

In summary, this study indicates that the E2-ferritin NPs induce superior protective immune responses to that of CV-E2. These NPs hold the potential to serve as an effective alternative vaccine for CSF, offering DIVA characteristics, and could play a significant role in the control and eradication of CSF.

## 5. Conclusions

In this study, we successfully developed a CHO suspension cell line that stably expresses the SpyTag-E2 protein and designed an E2-ferritin NPs vaccine using ferritin.

The in vivo experiments demonstrated that, compared to conventional subunit vaccines, including a commercial vaccine, the E2-ferritin NPs elicited robust cellular and humoral immune responses in pigs and provided complete protection against the lethal CSFV-SM.

## Figures and Tables

**Table 1:** Detection of the viral RNA in the blood samples from different groups by RT-qPCR.

Groups	Pig No.	Days Post-Challenge			
		3	7	10	14
E2-ferritin NPs	A1	-	-	-	-
	A2	-	-	-	-
	A3	-	-	-	-
	A4	-	-	-	-
SpyTag-E2	B1	-	-	-	-
	B2	-	-	-	-
	B3	-	-	-	-
	B4	-	-	-	-
CV-E2	C1	-	4.21 × 10^2^	-	-
	C2	-	2.60 × 10^3^	-	-
	C3	-	4.81 × 10^2^	-	-
PBS	D1	3.33 × 10^2^	2.06 × 10^3^	1.50 × 10^5^	/
	D2	2.36 × 10^3^	1.40 × 10^4^	2.30 × 10^6^	/

-: not detectable; /: dead.

**Table 2:** Detection of the viral RNA in the nasal samples from different groups by RT-qPCR.

Groups	Pig No.	Days Post-Challenge			
		3	7	10	14
E2-ferritin NPs	A1	-	-	-	-
	A2	-	-	-	-
	A3	-	-	-	-
	A4	-	-	-	-
SpyTag-E2	B1	-	-	-	-
	B2	-	-	-	-
	B3	-	-	-	-
	B4	-	-	-	-
CV-E2	C1	-	0.73 × 10^2^	-	-
	C2	-	0.96 × 10^2^	-	-
	C3	-	0.98 × 10^2^	-	-
PBS	D1	-	1.18 ×10^2^	1.33 × 10^3^	/
	D2	-	1.85 × 10^2^	1.39 × 10^3^	/

-: not detectable; /: dead.

**Table 3:** Detection of the viral RNA in the rectal samples from different groups by RT-qPCR.

Groups	Pig No.	Days Post-Challenge			
		3	7	10	14
E2-ferritin NPs	A1	-	-	-	-
	A2	-	-	-	-
	A3	-	-	-	-
	A4	-	-	-	-
SpyTag-E2	B1	-	-	-	-
	B2	-	-	-	-
	B3	-	-	-	-
	B4	-	-	-	-
CV-E2	C1	-	1.27 × 10^2^	-	-
	C2	-	1.55 × 10^2^	-	-
	C3	-	1.40 × 10^2^	-	-
PBS	D1	-	1.52 × 10^2^	6.70 × 10^3^	/
	D2	-	2.20 × 10^2^	6.48 × 10^3^	/

-: not detectable; /: dead.

**Table 4:** Detection of the viral RNA in the oral samples from different groups by RT-qPCR.

Groups	Pig No.	Days Post-Challenge			
		3	7	10	14
E2-ferritin NPs	A1	-	-	-	-
	A2	-	-	-	-
	A3	-	-	-	-
	A4	-	-	-	-
SpyTag-E2	B1	-	-	-	-
	B2	-	-	-	-
	B3	-	-	-	-
	B4	-	-	-	-
CV-E2	C1	-	0.97 × 10^2^	-	-
	C2	-	1.60 × 10^2^	-	-
	C3	-	1.07 × 10^2^	-	-
PBS	D1	-	1.27 × 10^2^	3.13 × 10^2^	/
	D2	7.37 × 10^2^	1.94 × 10^2^	9.36 × 10^3^	/

-: not detectable; /: dead.

## Data Availability

The data presented in this study are available on request from the corresponding author.
